# Venoarterial-extra corporeal membrane oxygenation-assisted parathyroidectomy for hypercalcemic crisis due to parathyroid carcinoma complicated by severe circulatory and respiratory failure: a case report

**DOI:** 10.1186/s40981-023-00606-y

**Published:** 2023-03-14

**Authors:** Yuria Enomoto, Yuko Matsuda, Yusuke Nagamine, Takahisa Goto

**Affiliations:** Department of Anesthesiology and Critical Care Medicine, Yokohama City University Hospital, 3-9 Fukuura, Kanazawa-Ku, Kanagawa 236-0004 Yokohama, Japan

**Keywords:** Hypercalcemic crisis, Hyperparathyroidism, Parathyroidectomy, Venoarterial-extra corporeal membrane oxygenation, VA-ECMO

## Abstract

**Background:**

Hypercalcemia crisis is a rare but severe form of hypercalcemia complicated by multiple organ failure. Hypercalcemia crisis due to hyperparathyroidism is commonly caused by a parathyroid tumor, which often requires surgical resection. However, there are no clear recommendations on when the surgery should be performed.

**Case presentation:**

A 64-year-old female patient developed hyperparathyroidism due to a parathyroid tumor and hypercalcemic crisis, which was complicated by severe circulatory and respiratory failure refractory to medical therapy, and an emergent surgery was planned to resect the parathyroid tumor. To prevent intraoperative circulatory and respiratory collapse, venoarterial-extra corporeal membrane oxygenation (VA-ECMO) was introduced, resulting in a safe operation and anesthetic management.

**Conclusions:**

In patients with hypercalcemic crisis complicated by severe circulatory and respiratory failure, induction of prophylactic VA-ECMO was useful for safe anesthetic management. Surgical resection should be performed as soon as the diagnosis is made before VA-ECMO is required.

## Background

Hypercalcemia crisis is a rare but severe form of hypercalcemia complicated by multiple organ failure. The most common cause of hypercalcemia crisis is primary hyperparathyroidism caused by a parathyroid tumor, which often requires surgical resection [1]. However, there are no clear recommendations on when the surgery should be performed. Although there have been case reports of venoarterial-extra corporeal membrane oxygenation (VA-ECMO) support after cardiac arrest due to hypercalcemia crisis [2], there have been no reports of prophylactic VA-ECMO for circulatory and respiratory failure during surgical treatment of hypercalcemia crisis. In this report, we describe a case in which prophylactic VA-ECMO was used to safely manage circulatory and respiratory failure during parathyroid tumor resection with hypercalcemic crisis due to parathyroid cancer. The patient’s informed consent was obtained for this case report. This manuscript adheres to the CARE guidelines [3].

## Case presentation

**Fig. 1:**
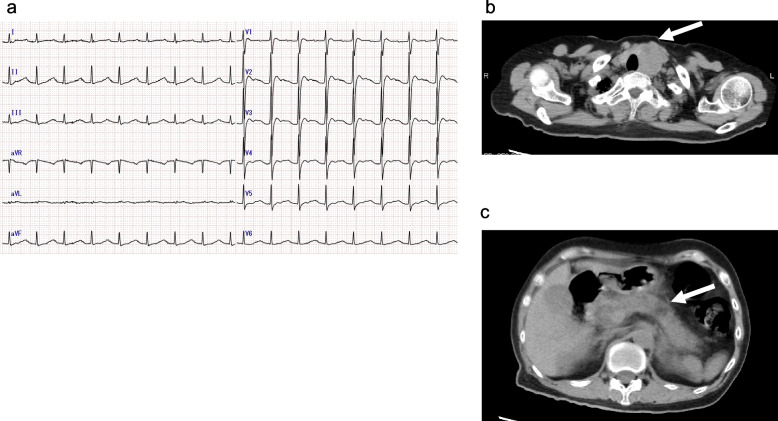
Electrocardiogram (**a**) and neck and abdominal CT images (**b** and **c**) on admission. Electrocardiogram demonstrated marked QT prolongation (corrected QT interval, 695 ms) (**a**). CT images showed a 40 × 30 mm mass on the left side of the thyroid gland (**b**). Enlarged pancreas with increased fat concentration (**c**) (white arrows)

**Fig. 2:**
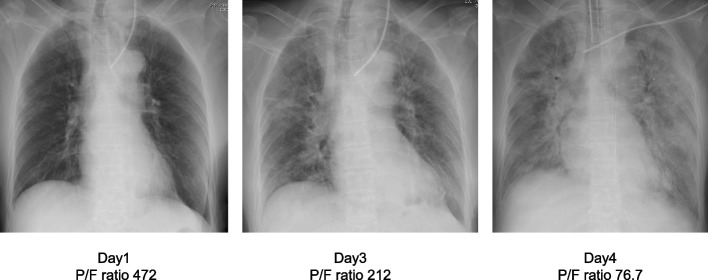
Chest radiograph, P/F ratio = PaO_2_/FiO_2_ ratio

A 64-year-old female (152 cm, 42.4 kg) with a history of ureteral stone was referred to our hospital for asymptomatic hematuria. She was alert, SpO_2_ was 94% in room air, and body temperature was 36.4˚C on admission. The blood pressure was 78/50 mmHg, and the heart rate were 100/min with sinus rhythm but marked QT prolongation (corrected QT interval, 695 ms) (Fig. 1a). Chest XP demonstrated no abnormal findings (Fig. 2), computed tomography (CT) showed a 40 × 30 mm mass on the left thyroid gland and an enlarged pancreas (Fig. 1b, c). Blood test was remarkable for calcium 21.9 mg/dL, phosphorus 4.6 mg/dL, potassium 2.2 mmol/L, magnesium 1.0 mg/dL, blood urea nitrogen 57 mg/dL, creatinine 2.42 mg/dL, and prothrombin time international normalized ratio of 1.44. Of note, serum lipase was 499 U/L (normal range: 14–54 U/L) and parathyroid hormone (PTH) was 2204 pg/mL (normal range: 15–65 pg/mL). She received fluid infusion and calcitonin with a diagnosis of hyperparathyroidism due to parathyroid tumor and secondary acute pancreatitis. Parathyroidectomy was planned after improvement of the general condition at this time.

**Fig. 3:**
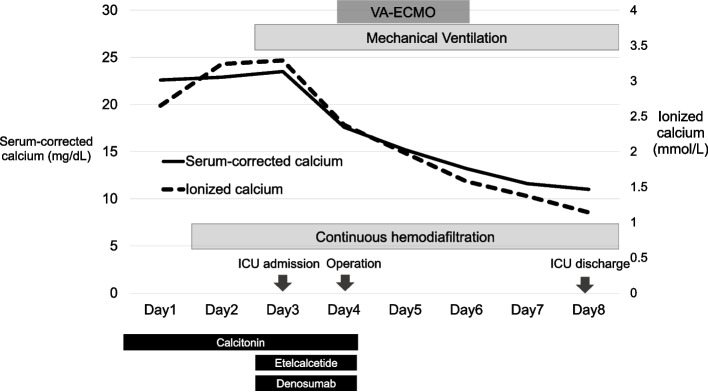
Clinical course, corrected, and ionized serum calcium concentrations. Serum-corrected calcium concentration (mg/dL) = measured calcium concentration (mg/dL) + 4-albumin (g/dL)

On day 2, continuous hemodiafiltration and infusion of noradrenaline 0.05 μg/kg/min was started for acute kidney injury, for remarkably increased serum calcium level of 22.3 mg/dL and persistent hypotension (Fig. 3). On day 3, the patient was endotracheally intubated and admitted to the intensive care unit (ICU) because of hypoxia with arterial partial oxygen pressure (PaO_2_)/fractional inspired oxygen concentration (FiO_2_) ratio of 212 mmHg due to acute respiratory distress syndrome (ARDS). Besides noradrenaline 0.2 μg/kg/min, adrenaline 0.04 μg/kg/min and vasopressin 0.02 unit/min, administration of etelcalcetide for regulating PTH secretion and denosumab, an anti-receptor activator of NF-κB ligand (RANKL) antibody for inhibiting osteoclast activation was started. The respiratory condition worsened further to a PaO_2_/FiO_2_ ratio of 76.7 mmHg with rapid progressing bilateral lung infiltrate shadow (Fig. 2). Emergent parathyroidectomy under VA-ECMO was planned for possible fatal arrhythmias caused by severe hypercalcemia resulting from surgical maneuver of the tumor on day 4.

General anesthesia was induced and maintained with propofol, fentanyl, and remifentanil with continuous infusion of adrenaline ≤ 0.05 μg/kg/min, noradrenaline ≤ 0.2 μg/kg/min, and vasopressin ≤ 0.03 units/min under monitoring of arterial pressure, central venous pressure, and transesophageal echocardiography. No intracardiac or pulmonary emboli were detected; continuous hemodiafiltration was continued intraoperatively. After insertion of drainage and return catheters to the right atrium through the right femoral vein and to the right femoral artery, respectively, ECMO was started at a flow rate of 3.5 L/min. Nafamostat mesilate was infused for maintaining activated coagulation time approximately 200 s. Surgical resection of the left parathyroid tumor and combined left lobe of thyroid were completed uneventfully. Duration of surgery and anesthesia was 177 and 284 min, respectively. Intraoperative blood loss was 202 mL, and we transfused 840 ml of red blood cells, 720 mL of fresh frozen plasma, and 200 mL of the platelet. The patient was returned to the ICU with ECMO support.

PTH was markedly decreased to 31 pg/mL, and the serum calcium level decreased to 15.2 mg/dL on postoperative day 1 (day 5). Vasoactive drug requirements were reduced in response to improved cardiac function, and the patient was weaned from ECMO on day 6. She was discharged from the ICU on day 8 with a serum calcium level of 11 mg/dL and an ionized calcium level of 1.14 mmol/L (Fig. 3). Pathological findings of the excised tumor suggested a parathyroid carcinoma. After discharge from the ICU, she developed further complications, including pneumothorax, ventilator-associated pneumonia, and sepsis associated with a relapse of pancreatitis, resulting in a long hospital stay. The patient was transferred to a rehabilitation hospital with a tracheostomy on day 104.

## Discussion

Hypercalcemic crisis is a rare but potentially fatal condition with multiple organ damage [1]. Its incidence is 6.7% among patients undergoing parathyroidectomy [4], and the most common cause is primary hyperparathyroidism by a parathyroid tumor [5]. Although no clear diagnostic criteria have been established, it is defined as a rapid increase in serum calcium level above 15 mg/dL, oliguria or elevated urea nitrogen, and acute onset of gastrointestinal, circulatory, or central nervous system symptoms [6].

Hypercalcemic crisis triggers numerous symptoms [1], from mild ones such as gastrointestinal symptoms, urinary tract stone, dehydration, muscular, and neurological symptoms to serious complications including cardiorespiratory failure requiring venovenous (VV)-ECMO [7] and VA-ECMO [2, 8]. Acute pancreatitis as observed in our case is more common in patients with severe crisis [4, 9]. It causes abnormal electrocardiogram and cardiac dysfunction. The action potential phase 2 shortens due to increased extracellular calcium concentration, and shortening of the ST and QT portions is common ECG findings. In our case, the ECG findings reflected a QT prolongation, fusion of *T* and *U* waves, and an enhancement of *U* wave, observed in hypokalemia. Intracellular hypercalcemia causes impaired diastolic relaxation due to impaired myocardial repolarization [10], and myofibrillar hypercontraction and subsequent myocardial necrosis [11].

Hypercalcemic crisis patients with impaired cardiac function should be monitored for the development of intracardiac thrombi. Chan et al. reported a case of intracardiac thrombi in a patient with hypercalcemic crisis under VA-ECMO [8]. Hyperparathyroidism and hypercalcemia have been reported to induce activation of coagulation factors and increased platelet aggregation, which may trigger various type of thrombosis [12]. Hypercalcemic crisis in patients with an impaired cardiac function requiring VA-ECMO may be more susceptible to intracardiac thrombosis due to procoagulant stimuli by exposure to ECMO circuits [8], requiring strict monitoring for intracardiac thrombosis.

In this case, two potential etiologies are postulated as causes of respiratory failure and ARDS. First, pulmonary edema due to infusion overload for hypercalcemia symptoms. Infusion overload due to acute renal failure or acute pancreatitis may also act in an additive manner. Second, animal studies have shown that hypercalcemia causes pulmonary edema via activation of inducible nitric oxide synthase and increased nitric oxide production and inflammatory cytokines [13].

There is much debate regarding the timing of surgical treatment in cases of hypercalcemic crisis, varying from emergency to elective surgery after correction of electrolytes [1, 5, 6], although the timing of surgery was not associated with the long-term prognosis [14]. In our case, the patient’s symptoms progressed very rapidly, and the tumor resection was performed when the patient’s circulatory and respiratory status had collapsed. If the surgery had been performed at the initial stage, a non-invasive and safe anesthetic management could have been performed before the progression to multiple organ failure. In cases of the severe form of hypercalcemic crisis complicated by circulatory and respiratory failure, we believe that urgent surgical intervention should be performed as soon as the diagnosis is made.

In conclusion, we experienced parathyroid tumor resection in a patient with hypercalcemic crisis complicated by severe circulatory and respiratory failure. We performed the surgery after introducing prophylactic VA-ECMO for intraoperative circulatory and respiratory collapse. In patients with the most severe form of hypercalcemic crisis, surgical resection should be performed as soon as the diagnosis is made.

## Data Availability

Not applicable.

## Abbreviations

ARDS: Acute respiratory distress syndrome
CT: Computed tomography
ICU: Intensive care unit
FiO_2_: Fractional inspired oxygen concentration
PaO_2_: Arterial partial pressure of oxygen
P/F ratio: PaO_2_/FiO_2_ ratio
PTH: Parathyroid hormone
RANKL: Receptor activator of NF-κB ligand
VA-ECMO: Venoarterial-extra corporeal membrane oxygenation
VV-ECMO: Venovenous-extra corporeal membrane oxygenation
